# Extending Cardiac Functional Assessment with Respiratory-Resolved 3D Cine MRI

**DOI:** 10.1038/s41598-019-47869-z

**Published:** 2019-08-09

**Authors:** Jing Liu, Yan Wang, Zhaoying Wen, Li Feng, Ana Paula Santos Lima, Vaikom S. Mahadevan, Ann Bolger, David Saloner, Karen Ordovas

**Affiliations:** 1 Department of Radiology and Biomedical Imaging, University of California San Francisco, San Francisco, California United States; 2 Department of Radiology, Anzhen Hospital, Capital Medical University, Beijing, China; 3 Department of Medical Physics, Memorial Sloan Kettering Cancer Center, New York, NY United States; 4 Department of Cardiology, University of California San Francisco, San Francisco, California United States; 5 Radiology Service, VA Medical Center, San Francisco, California United States

**Keywords:** Three-dimensional imaging, Magnetic resonance imaging

## Abstract

This study aimed to develop a cardiorespiratory-resolved 3D magnetic resonance imaging (5D MRI: x-y-z-cardiac-respiratory) approach based on 3D motion tracking for investigating the influence of respiration on cardiac ventricular function. A highly-accelerated 2.5-minute sparse MR protocol was developed for a continuous acquisition of cardiac images through multiple cardiac and respiratory cycles. The heart displacement along respiration was extracted using a 3D image deformation algorithm, and this information was used to cluster the acquired data into multiple respiratory phases. The proposed approach was tested in 15 healthy volunteers (7 females). Cardiac function parameters, including the end-systolic volume (ESV), end-diastolic volume (EDV), stroke volume (SV), and ejection fraction (EF), were measured for the left and right ventricle in both end-expiration and end-inspiration. Although with the proposed 5D cardiac MRI, there were no significant differences (*p* > 0.05, t-test) between end-expiration and end-inspiration measurements of the cardiac function in volunteers, incremental respiratory motion parameters that were derived from 3D motion tracking, such as the depth, expiration and inspiration distribution, correlated (*p* < 0.05, correlation coefficient, Mann-Whitney) with those volume-based parameters of cardiac function and varied between genders. The obtained initial results suggested that this new approach allows evaluation of cardiac function during specific respiratory phases. Thus, it can enable investigation of effects related to respiratory variability and better assessment of cardiac function for studying respiratory and/or cardiac dysfunction.

## Introduction

Cardiac magnetic resonance imaging (CMR) has been considered the gold standard for quantification of heart volume and function^1,2^. On one hand, breath-hold cardiac cine acquisitions are routinely used in daily clinical protocols. However, the respiratory position at which data are acquired is highly dependent on scan protocols and the patients’ capabilities to hold their breath. For example, data may be acquired at either an end-inspiratory or end-expiratory phase depending on breathing instructions given by an operator. On the other hand, although cardiac images can also be acquired during free breathing, a specific cardiac phase may be acquired at either expiration or inspiration. This lack of standardization can introduce significant variability in the measures of cardiac volumes and function, which are particularly relevant when serial CMR studies are used to monitor cardiac dysfunction, in those patients with congenital heart disease, cardiomyopathies, and valvular lesions^3–5^. Respirophasic changes in venous return^6^ and afterload predominantly affect the right side of the heart, although those respiratory events have less effect on the left ventricular filling and output under normal conditions. Thus, utilization of a single time point in the respiratory cycle, such as end-expiration, creates an artificial construction that may obscure physiologically important variability in the function of both left ventricle (LV) and right ventricle (RV), particularly in diseases such as pulmonary hypertension or constrictive pericarditis that present with abnormal systemic venous return and RV afterload.

Respiratory-resolved cardiac functional assessment is a promising approach to overcome the abovementioned limitations. However, its main challenges include lengthy scan times and potential inaccuracies in measurements due to cardiac and respiratory motion. Several studies have previously proposed novel imaging methods for evaluating the effects of respiratory motion on cardiac function, but most of them were based on 2D cardiac MRI^7–9^, which may suffer from slice misregistration errors. Cardiorespiratory-resolved 3D, or termed as 5D MRI, is a desired imaging technique and has the potential to address the challenge of slice misregistration. For example, Sigfridsson and colleauges^10^ have previously proposed a 5D MRI approach incorporating a cardiac motion and a respiratory motion dimension. However, management of physiological motion and long scan times still remain as big challenges for 5D MRI towards routine clinical use. Respiratory motion tracking can be done by using bellows or derived self-gating signals during continuous data acquisitions, which usually provide either relative or absolute respiratory motion tracking^11–16^. However, these approaches normally provide motion information along one dimension only, and multiple studies have suggested that motion tracking in three dimensions in an absolute fashion allows more accurate gating of acquired data or motion correction^17,18^. Here, acquisitions of 3D navigation data are desirable, but it is often time-consuming and can be challenging to combine with cine imaging.

Compressed sensing has become a powerful approach for rapid MRI, and 5D MRI is an ideal candidate for the application of compressed sensing methods due to its extensive correlations along both cardiac and respiratory dimensions. In order to achieve efficient selection of k-space data points for compressed sensing MRI, a number of undersampling strategies have been developed for continuously interleaved data acquisitions, and many of them can be implemented directly on a Cartesian grid^19–24^ to provide favorable features of incoherent undersampling while minimizing the sensitivities to eddy current, offer-resonance effect, gradient delay, and some other effects presented in non-Cartesian trajectories. One of such underudersampling schemes, known as CIRcular Cartesian UnderSampling (CIRCUS)^25^, has previously been demonstrated for several cardiac MRI applications, allowing pseudo-random variable-density and interleaving features for highly accelerated dynamic MRI^16,26,27^.

In this study, we aimed to combine CIRCUS with a multicoil compressed sensing method (k-t SPARSE-SENSE) for a new highly-accelerated 5D MR imaging protocol that can be used for free-breathing cardiac functional assessment in ~2.5 minutes. We also implemented a 3D motion detection algorithm to track the movement of the heart along respiration, which is needed to guide data sorting during reconstruction. The feasibility of the proposed 5D cardiac cine MRI method was demonstrated for assessment of cardiac function in normal volunteers by comparing quantification between end-expiration and end-inspiration and investigating the influence of respiration on the measurements of cardiac function.

## Results

**Figure 1:**
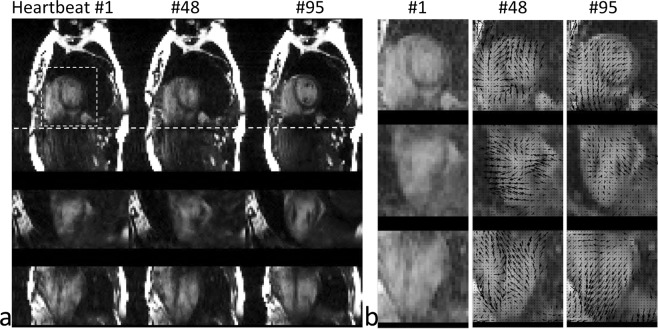
(**a**) 3D images with a low spatial resolution (4 × 4 × 4 mm^3^) reconstructed for motion tracking at 3 representative heartbeats. (**b**) Corresponding deformation vector fields from heartbeats #48 and #95 relative to the reference heartbeat #1 are shown with black arrows.

**Figure 2:**
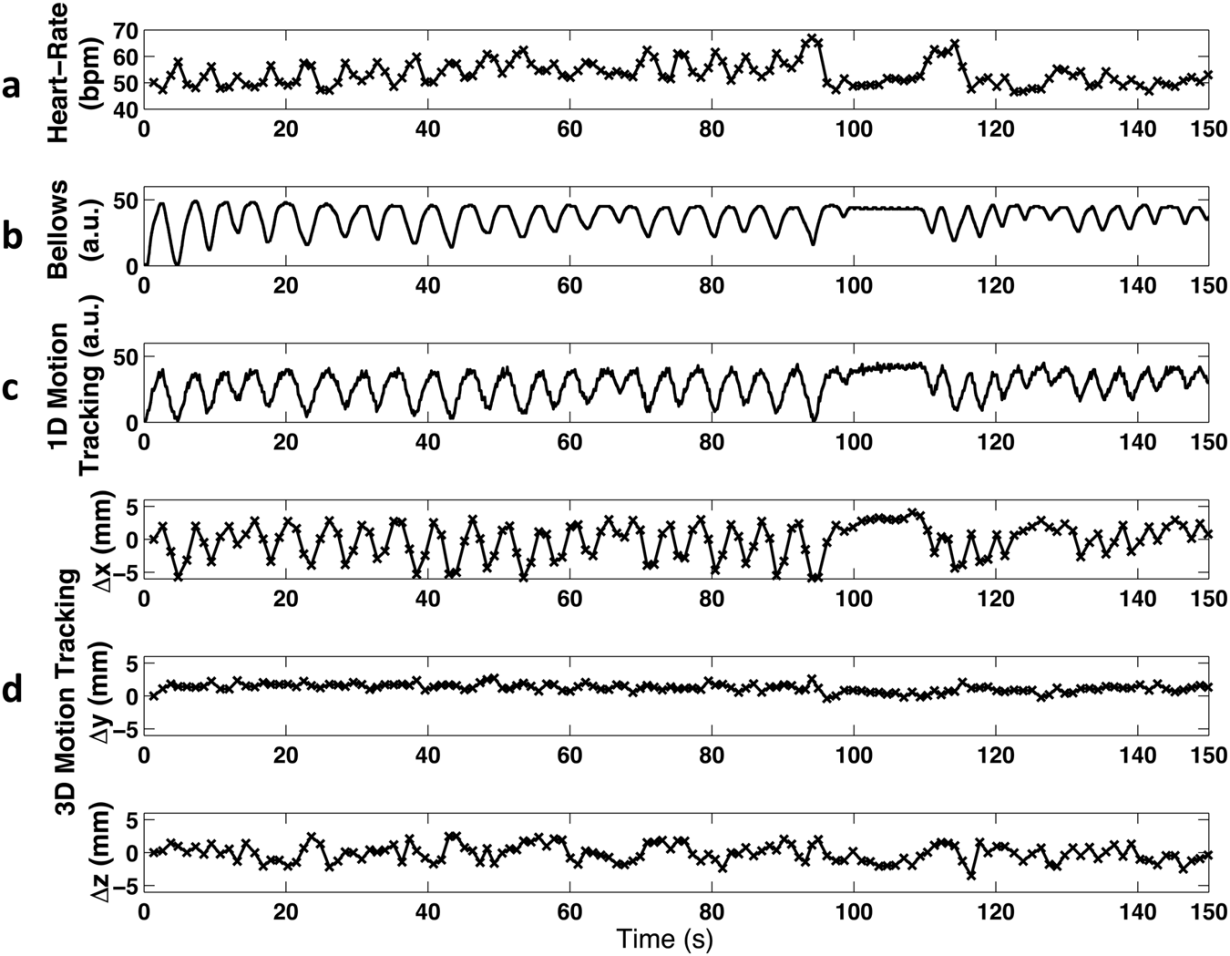
(**a**) Instantaneous heart rate, (**b**) bellows signal, (**c**) 1D self respiratory gating signal and (**d**) 3D motion tracking from a healthy volunteer during a scan of 2.5 minutes.

In Fig. 1a, low spatial resolution images for motion tracking selected from three representative heartbeats were reformatted and shown in three orthogonal planes. The deformation motion fields between the target images (heartbeats #48 and #95) and the reference (heartbeat #1) are displayed as black arrows and are superimposed on the central region of the images in Fig. 1b. The directions of the arrows indicate the deformation vector fields pointing from the current time frame back to the reference frame. We measured the average deformation in three directions within a central region, covering 1/4 field of view (FOV) around the center (white box with dashed line edges in Fig. 1a), per heartbeat throughout the entire scan, as shown in Fig. 2d. The instantaneous heart rate (Fig. 2a), bellows gating (Fig. 2b), and 1D self-gating signal^16^ (Fig. 2c) are also plotted for comparison.

The correlation coefficient between the 1D and 3D motion signals derived from the 15 volunteer data sets was 0.81 ± 0.18 (p < 0.001, significant correlation), and the dice coefficient of the clustering of the two motion signals was 0.68 ± 0.12. The data assigned for EE (end-expiration) and EI (end-inspiration) phases were 31.8 ± 9.8% and 20.7 ± 5.3% respectively with 3D motion tracking and clustering, which were not significantly different from 28.4 ± 19.8% (p = 0.56) and 23.8 ± 15.4% (p = 0.46) with 1D motion tracking and clustering. LV cardiac functional measurements from both 3D and 1D motion tracking methods were shown to be comparable to those acquired with 2D cine MRI, as shown in Table 1.

**Table 1:** Left ventricular volume-based measurements with 2D cine and 5D MRI using 1D and 3D motion tracking.

	ESV (mL)	EDV (mL)	SV (mL)	EF (%)
2D Imaging - EE	45.4 ± 13.4	120.7 ± 28.2	75.3 ± 18.3	62.5 ± 5.6
5D Imaging - 1D Motion Tracking - EE	49.4 ± 15.7	126.0 ± 27.2	72.7 ± 14.9	59.9 ± 5.3
Bias (vs 2D)	4.0 ± 6.3	1.4 ± 11.4	−2.6 ± 8.9	−2.6 ± 4.0
p-value (vs 2D)	0.53	0.91	0.72	0.28
5D Imaging - 3D Motion Tracking - EE	48.1 ± 14.5	123.3 ± 27.1	75.2 ± 15.1	61.3 ± 4.8
Bias (vs 2D)	2.7 ± 6.4	2.6 ± 6.1	−0.1 ± 7.8	−1.1 ± 5.2
p-value (vs 2D)	0.66	0.83	0.99	0.62
Bias (vs 1D motion tracking)	−1.3 ± 6.0	1.2 ± 8.5	2.5 ± 6.9	1.5 ± 3.9
p-value (vs 1D motion tracking)	0.84	0.92	0.70	0.51
5D Imaging - 1D Motion Tracking - EI	52.1 ± 17.2	124.2 ± 26.8	70.7 ± 11.9	58.3 ± 5.2
5D Imaging - 3D Motion Tracking - EI	49.4 ± 15.2	122.6 ± 25.2	73.1 ± 14.3	60.1 ± 6.3
Bias (vs 1D motion tracking)	−2.7 ± 7.4	−0.24 ± 9.6	2.4 ± 7.6	1.8 ± 4.9
p-value (vs 1D motion tracking)	0.84	0.95	0.74	0.60

The differences of the measurements between 3D and 1D motion tracking methods or between 3D and 2D cine MRI methods are not significant (significance level of 0.05, N = 11).

ESV: end-systolic volume; EDV: end-diastolic volume; SV: stroke volume; EF: ejection fraction; EE: end-expiration; EI: end-inspiration.

**Figure 3:**
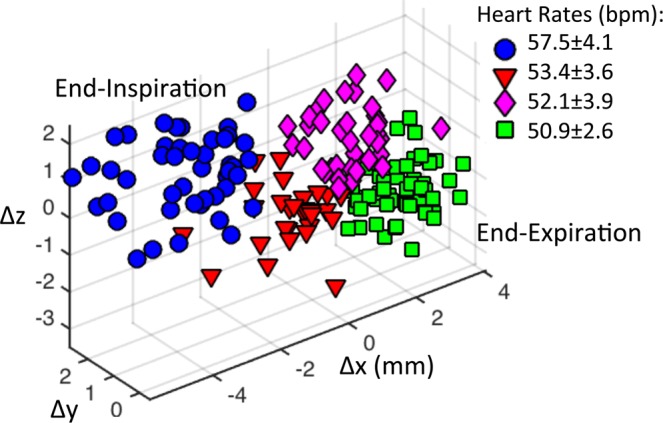
Clustering of 3D respiratory motion to different respiratory phases in a representative volunteer. Each data point corresponds to the 3D motion tracking at one heartbeat. The four respiratory phases are displayed with different colors and shapes: end inspiration, blue circles; expiratory, red triangles; end-expiration, green squares; inspiratory, pink diamonds. The averaged instantaneous heart-rate at each respiratory phase was calculated.

Figure 3 shows the 3D plot of the motion distribution in three directions, which were clustered into four groups using a k-means clustering algorithm, representing end-expiration phase (green squares), end-inspiration phase (blue circles) and the two phases in-between. In Fig. 3, the displacement along x refers to the motion along the vertical axis of the short-axis view images (Fig. 1), where the positive displacement corresponds to how much the heart moves up (the diaphragm moves up correspondingly). In this study, the displacements of the heart along x direction has been used as a guidance for assigning EE and EI phases (Fig. 3), where the cluster with the most positive displacements along x was defined as EE and the one with the most negative displacements along x was defined as EI. The average instantaneous heart-rates at different respiratory phases were calculated, showing a lower heart rate at end-expiration (50.9 ± 2.6 bpm) and a higher rate at end-inspiration (57.5 ± 4.1 bpm), due to respiratory sinus arrhythmia (RSA)^28^. This variation in the heart rate can be clearly observed in the heart rate curve shown in Fig. 2a.

**Figure 4:**
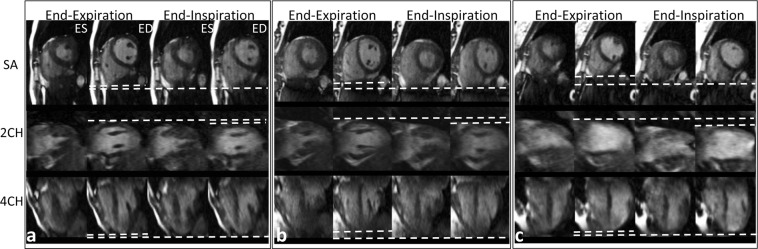
Cardiac images from three subjects with different respiratory depths (left: Δd = 3.42 mm; middle: Δd = 6.23 mm; right: Δd = 12.60 mm). ES: end-systole, ED: end-diastole, SA: short-axis view, 2CH: two chamber view, 4CH: four chamber view. The dotted lines highlight the variation of respiratory depths in different subjects along three orthogonal directions, mainly in Superior to Inferior (S/I) direction.

Figure 4 shows images from three representative cases with different respiratory motion scale. With 3D motion tracking, we could measure the average displacement (respiratory depth, Δd) of the heart between end-expiration and end-inspiration during the scan. The dotted lines in Fig. 4 show the movement of the heart in three directions.

The proposed 3D motion tracking allows respiration related measurements, as reported in Table 2. End-expiration has a larger percentage of the measurements compared to end-inspiration (31.8 ± 10.2% vs 20.7 ± 5.5%, *p* = 0.001, significant), while end-expiration has about 30% smaller average motion (1.9 ± 1.3 vs 2.8 ± 2.6 mm, *p* = 0.25, not significant). We also noticed that the male subjects have a larger portion of data at expiration compared to the female subjects (35.1%; interquartile range 30.8–46.9% vs 24.8%; 22.5–31.0%) (*p* = 0.03, significant).

**Table 2:** Measurements related to respiratory motion. Mean and standard deviation are reported.

	Respiratory Rate (brpm)	Respiratory Depth (mm)	Heart Rate (bpm)	Data Portion (%)	Average Motion (mm)	Data Ratio (EE to EI)
All (N = 15)	14.5 ± 4.7	5.3 ± 2.3	65.4 ± 8.4			1.6 ± 0.5
EE			64.0 ± 8.5	31.8 ± 9.8*	1.9 ± 1.3	
EI			67.2 ± 8.1	20.7 ± 5.3*	2.8 ± 2.5	
Females (n = 7)	16.5 ± 5.4	6.0 ± 2.9	66.7 ± 10.1			1.4 ± 0.5
EE			64.7 ± 10.4	25.6 ± 4.8**	1.6 ± 0.8	
EI			68.4 ± 9.5	19.8 ± 5.9	2.1 ± 0.7	
Males (n = 8)	12.8 ± 3.1	4.7 ± 1.4	64.3 ± 6.3			1.8 ± 0.5
EE			63.4 ± 6.3	37.2 ± 9.8**	2.2 ± 1.5	
EI			66.0 ± 6.5	21.5 ± 4.6	3.4 ± 3.2	

The differences of the paired measurements between two respiratory phases are not significant except those highlighted with *(p = 0.001 calculated using t-test), and the significant differences between two genders are highlighted with **(p = 0.03 calculated using Mann-Whitney test), based on significance level of 0.05.

EE: end-expiration; EI: end-inspiration; Respiratory Depth: the three-dimensional displacement (Δd) of the heart between EE to EI; Data Portion: the percentage of the data from the entire respiratory cycle that occurs in EE or EI; Average Motion: the averaged distance of the clustered data points from the center of the cluster in EE or EI; Data Ratio (EE to EI): the ratio between the data portions (by counts of heartbeats) at EE and EI; brpm: breaths per minute; bpm: beats per minute.

Table 3 summarizes the data analysis on the cardiac functional measurements at end-expiration and end-inspiration. All the measurements between two respiratory phases or two genders were not significantly different (*p*-values were greater than 0.05). The changes of the RV measurements between two respiratory phases were found without significance either, but they were much larger compared to those in the LV measurements, such as >8% vs <3% changes in ESV, EDV and SV (Table 3).

**Table 3:** Left and right ventricular volume-based measurements at end-expiration and end-inspiration.

LV	ESV (mL)	EDV (mL)	SV (mL)	EF (%)
All (N = 15)				
EE	47.0 ± 13.2	123.2 ± 24.7	76.2 ± 13.6	62.2 ± 4.4
EI	48.1 ± 13.2	122.2 ± 23.6	74.2 ± 14.1	60.9 ± 5.4
Change (%)	2.9 ± 7.7	−0.6 ± 2.2	−2.7 ± 6.9	−2.1 ± 5.4
*p*-value	0.83	0.91	0.69	0.48
Females (n = 7)				
EE	42.9 ± 5.9	113.8 ± 14.3	70.9 ± 12.1	62.2 ± 4.5
EI	44.2 ± 7.1	113.1 ± 13.5	68.9 ± 13.0	60.7 ± 6.1
Change (%)	2.9 ± 7.0	−0.5 ± 2.2	−2.8 ± 7.2	−2.4 ± 5.8
*p*-value	0.73	0.93	0.79	0.65
Males (n = 8)				
EE	50.6 ± 16.3	131.5 ± 28.6	80.9 ± 13.1	62.3 ± 4.4
EI	51.5 ± 16.0	130.3 ± 27.3	78.7 ± 13.4	61.1 ± 4.7
Change (%)	2.8 ± 8.3	−0.7 ± 2.3	−2.6 ± 6.6	−1.9 ± 5.1
*p*-value	0.92	0.94	0.76	0.61
**RV**	**ESV (mL)**	**EDV (mL)**	**SV (mL)**	**EF (%)**
All (N = 15)				
EE	83.8 ± 18.6	156.1 ± 36.3	72.3 ± 21.3	46.0 ± 5.5
EI	90.6 ± 19.0	176.7 ± 45.6	86.1 ± 30.4	48.0 ± 5.6
Change (%)	8.8 ± 11.1	12.9 ± 9.9	19.1 ± 20.5	5.0 ± 12.1
*p*-value	0.32	0.18	0.16	0.33
Females (n = 7)				
EE	76.5 ± 17.3	142.5 ± 32.8	66.1 ± 21.0	45.9 ± 6.9
EI	79.6 ± 18.3	152.3 ± 36.2	72.7 ± 20.4	47.6 ± 4.8
Change (%)	4.4 ± 8.6	7.1 ± 8.1	12.4 ± 19.3	4.6 ± 12.2
*p*-value	0.74	0.60	0.56	0.62
Males (n = 8)				
EE	90.1 ± 18.3	167.9 ± 36.9	77.8 ± 21.4	46 ± 4.4
EI	100.3 ± 14.5	198.0 ± 43.9	97.8 ± 33.9	48.3 ± 6.6
Change (%)	12.7 ± 12.1	18.1 ± 8.6	25.0 ± 21.0	5.4 ± 12.8
*p*-value	0.23	0.16	0.18	0.42

Mean and standard deviation are reported. Differences of the measurements between end-expiration and end-inspiration (t-test, significance level of 0.05) or between females and males are not significant (Mann-Whitney test, significance level of 0.05). No significant differences were found between the respiratory phases or genders. The stroke volumes of LV and RV are not significantly different (*p*-value of 0.55 for end-expiration, *p*-value of 0.18 for end-inspiration).

ESV: end-systolic volume; EDV: end-diastolic volume; SV: stroke volume; EF: ejection fraction; EE: end-expiration; EI: end-inspiration; change = (EI-EE)/EE × 100.

We evaluated the correlations between cardiac functional measurements and respiration-related parameters, including the respiratory rate, respiratory depth, data portions at end-expiration and end-inspiration, respectively, and their ratios. Strong correlations (*p* < 0.05) were found between the LV measurements and the respiratory parameters. However, no significant correlations were found between the RV measurements and the respiratory parameters. In the healthy volunteer subjects, the end-inspiration portion correlates significantly (R = 0.61, *p* = 0.02; R = 0.56, *p* = 0.03) with left ventricle ejection fraction (LVEF) at end-expiration and end-inspiration respectively. Based on separate analyses of data from females and males, we found that in male subjects, the respiratory rate, depth and data portion at end-expiration had strong relationships with the cardiac functional measurements either at end-expiration or end-inspiration (such as R = 0.72 and R = 0.84 between SV and respiratory rate at end-expiration and end-inspiration respectively). Female subjects, on the other hand, had strong correlations between end-inspiration related respiratory parameters (end-inspiration portion, R = 0.82, and end-expiration-to-inspiration ratio, R = −0.87) and the LVEF at end-expiration. Changes of the cardiac measurements between end-expiration and end-inspiration were found to have no correlation with the respiratory parameters, when all cases or female subjects were evaluated, while several significant correlations were found in male subjects.

## Discussion

Cardiorespiratory interactions and their impact on variations in cardiac volumes and function during a respiratory cycle are well-described phenomena. This physiologic response can vary significantly among individuals, and in different health and disease states^29,30^. Respiratory variability during the cardiac cycle can also be used as a diagnostic tool. Changes in septal motion and configuration during the cardiac cycle are reflective of ventricular interaction^7,31^, and have been described as diagnostic features of constrictive pericarditis, cardiac tamponade, and pulmonary hypertension.

In this study, we have developed a rapid cardiorespiratory 3D cardiac MRI method for measuring cardiac function parameters at different respiratory phases. We have found interesting relationships between respiratory motion parameters and cardiac volume-based parameters. Although no significant differences in the cardiac measurements were found between end-expiration and end-inspiration based on the volunteer data, the differences are expected to be larger in patients with rhythm or ventricular load abnormalities. As a result, 5D MRI may allow better assessment of overall cardiac performance and the anticipated hemodynamic changes in conditions with increased variability in load and output during respiratory events, including congenital heart diseases, such as after Fontan procedure^32^, and combined respiratory and cardiac disorders^33^. Future investigation of our proposed 5D MRI in larger cohorts of patients will be required to confirm these initial observations and demonstrate its potential for clinical use.

The motion tracking algorithm implemented in this work enables measurements of absolute displacement of the heart during respiration in three spatial dimensions and is expected to provide more accurate sorting of the acquired data to different motion phases for motion-resolved image reconstruction. To initiatively demonstrate the benefit of using 3D motion tracking instead of 1D for respiratory binning, we have added simulations (Supplement A), demonstrating that 3D motion tracking provided more accurate data clustering of respiratory phases compared to the 1D motion tracking especially under thoracoabdominal asynchrony. However, view sharing was applied to the data throughout heartbeats to improve the image reconstruction that had a high undersampling factor. Although we found out that the 3D motion tracking achieved in this study was quite reliable with the images obtained with this reconstruction scheme, their image quality could be potentially improved by with more advanced image reconstruction techniques, such as parallel imaging and/or compressed sensing. To minimize temporal smoothing effect caused by image reconstruction with a temporal constraint, we have reconstructed 3D cine images at each respiratory phase separately. A joint 5D image reconstruction algorithm^34^ could also be applied for further improvement of image quality with optimized additional regularization parameter.

We have done simulations to test the performance of applying 3D motion tracking method with different spatial resolutions and motion scales. As reported in Supplement B, for simulated phantom images of 1 × 1 mm resolution, 4 × 4 mm resolution motion tracking could track the motion well for either relatively large (−8~8 mm) or small (−2~2 mm) motion scales. Previous studies on 3D motion detection^17,18^ showed that a relatively low-resolution image (4.4 mm) could be used to derive smaller displacements and to correct higher-resolution images (1.2 mm).

Preliminary results obtained from patients with arrhythmia or hypertension were also investigated to demonstrate the feasibility of the proposed 5D MRI towards routine clinical use (Supplement C).

Ejection fraction is an important cardiac function parameter to measure in order to study various cardiac disorders. We have investigated the relationships between LVEF and the respiratory parameters. For male subjects, the LVEF measured at end-expiration positively correlates with end-expiration portion (R = 0.83, *p* = 0.01), however, the female subjects had LVEF measured at end-expiration correlated with end-inspiration portion (R = 0.82, *p* = 0.02). Respiratory depth is strongly correlated with LVEF at end-expiration in males (R = 0.78, *p* = 0.02). We also found the change of LVEF between two phases was correlated with the respiratory depth in males (R = −0.74, *p* = 0.04). A study with a larger cohort is desired to have a more comprehensive evaluation on the effect of respiration on cardiac function between genders.

We acknowledge several limitations in this study. First, as recognized growing interest in generalizability of biomedical research depends on the consideration of key biological variables such as sex, we have investigated the gender difference in the respiratory and cardiac parameters we acquired. However, the sample size for each gender was small. Studies of larger cohorts are desired. Ideally a longer time scan would allow pursuing sufficient data to analyze the transition phases between end-expiration and end-inspiration and measuring the cardiac function throughout the entire respiratory cycle. Second, the conventional breath-hold 2D cine MRI used in our study was only performed during end-expiration. The data would be more complete if images could also be acquired during end-inspiration. Third, a study^9^ using real-time 2D MRI showed larger changes in LV volumes between expiration and inspiration, although we have observed a variation in the changes and the averaged change was small (Table 3). Since both that study and our own were based on a relatively small number of subjects, a study with a larger cohort is needed for a more comprehensive evaluation. Fourth, image reconstruction based on parallel imaging and compressed sensing techniques resulted in about two hours processing time per case in this study, which needs to be significantly improved for clinical use.

Compared to other cardiac cine imaging studies at 3 T, we achieved much thinner slice thickness (4–5 mm vs 8–10 mm), used a relative higher flip angle (60° vs 45°), and the gradient of the scanner has a relatively slower maximum slew rate of 120 mT/m/ms (vs 150–200 mT/m/ms), which resulted in a relatively longer TR of 4–4.4 ms. Longer TR used in bSSFP makes it more sensitive to field inhomogeneities, especially at 3 T. Carefully performing a local shim on the heart is critical for cardiac imaging, which we have applied in all of our scans to reduce the local inhomogeneities. Further reducing of TR duration is desired, which could be achieved by using improved gradient or RF pulse design.

5D XD-GRASP has many nice features and focuses on providing comprehensive morphological and functional assessment of the heart. However, it requires long scan times (>10 mins), high flip angle (~90 degrees) in order to see the coronary arteries without contrast, and a gridding step for reconstruction of the radial data. In this work, we focused more on delivering a fast, efficient and more practical method for measurement of myocardial function, which combined compressed sensing and parallel imaging with a Cartesian sequence. We also assessed the effect of respiratory motion on the heart, and investigated both cardiac and respiratory parameters and their relationships.

In summary, we have demonstrated our proposed cardiorespiratory-resolved 3D MRI by exploiting 3D motion tracking for evaluating cardiac function during respiration. This 5D MRI method has the potential to allow further understanding the effects of respiration on the cardiac function in health and disease, and is expected to provide a more accurate tool for assessment of cardiac function in the setting of combined cardiopulmonary disease.

## Methods

**Figure 5:**
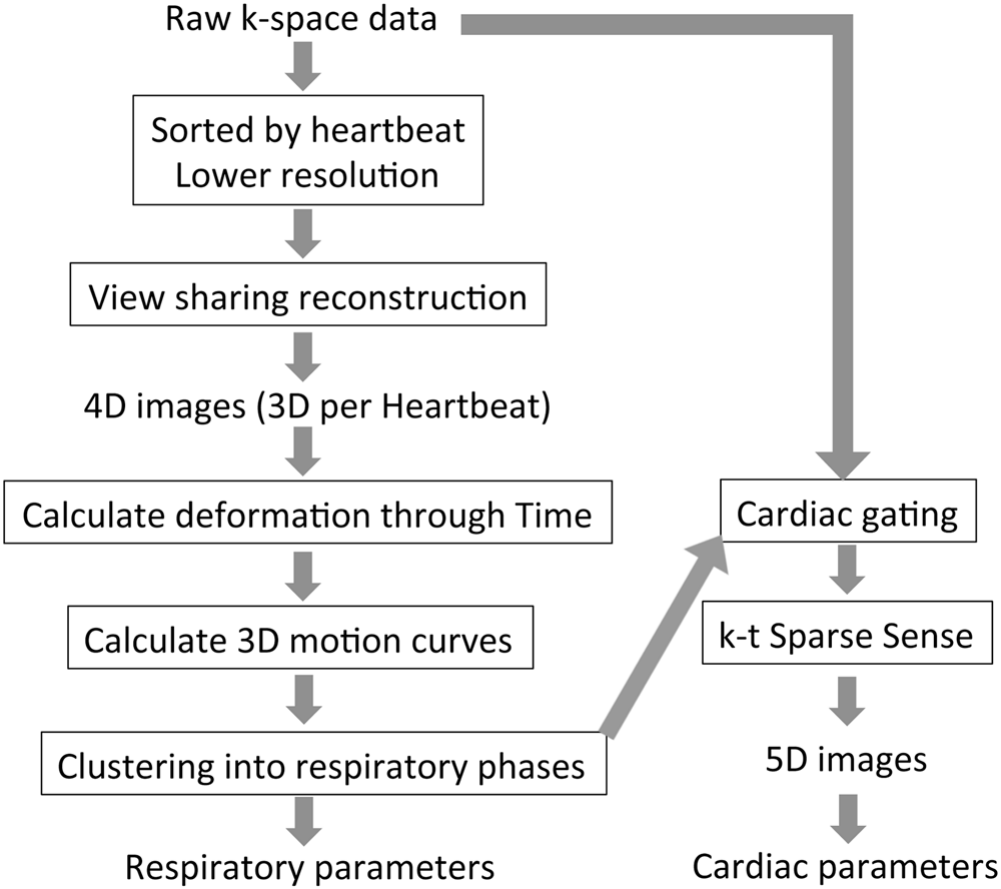
The pipeline of our data acquisitions, data pre-processing, image reconstruction and image analyses.

The pipeline of our data acquisitions, data pre-processing, image reconstruction and image analyses are summarized in Fig. 5 and are described in the following subsections. All methods were performed in accordance with the relevant guidelines and regulations.

### Free-breathing 3D cardiac MRI

As demonstrated previously in^16^, a continuous data acquisition with retrospective respiratory-gating has been explored for generating 3D cardiac cine MR images during free breathing. A similar data acquisition scheme was applied in this study. Instead of choosing (gating) data at a specific respiratory position (e.g., end-expiration) for 4D (3D cine) MRI reconstruction, we aimed to explore the continuously acquired data for 3D motion tracking in an absolute measurement (in units of mm) and reconstruct a 5D (cardiorespiratory motion-resolved 3D) image-set. During MR scans, electrocardiograph (ECG) triggers were saved and were retrospectively synchronized with the acquired image data (by readout lines).

### 3D Respiratory motion tracking derived from continuous data acquisition

The continuously acquired cardiac data were first sorted as different heartbeats using the recorded ECG triggers (i.e., data acquired in each heart-beat were reconstructed as a 3D image volume). By combining the data throughout each cardiac cycle, one can mitigate cardiac motion (significant part of the data is from the diastole), such that the motion between different 3D image volumes was mainly caused by respiration. The undersampling factor (R) for each of these 3D image volumes was ~17 (e.g. field of view (FOV) = 34 × 25.5 cm^2^, slice thickness = 4 mm, time of repetitions (TR) = 4 ms, image matrix = 256 × 144 × 30, heart rate = 60 bpm). In order to improve image quality, images were reconstructed with a lower spatial resolution (~4 × 4 × 4 mm) by using only the low frequency k-space region, resulting in an undersampling factor of R = 7.5. In addition, view sharing^35,36^ (nearest preference, mainly sharing data at high frequencies) was also applied to reduce undersampling effects.

Deformation fields between the 3D images in different heartbeats were derived by applying a similarity measure (e.g. the mutual information) between their image intensities^37^. Given that different organs actually exhibit different deformation during the respiration (e.g. the chest wall has a very different motion pattern compared to the heart), a central region of the image that mainly covers the heart (our organ of interest) was automatically chosen for calculating the averaged deformation for the motion tracking (absolute displacement) in three orthogonal directions.

### Respiratory phases binned by k-means clustering

The respiratory cycle is a mechanical process that depends on volume changes in the thoracic cavity and the corresponding diaphragm movements cause the motion of the heart during respiration. Respiration includes expiration and inspiration. End-inspiration can be defined as maximal diaphragmatic excursion whereas end-expiration defined as minimal diaphragmatic excursion. The periods between these two phases are mid-expiration and mid-inspiration^38^. In this study, we divided the respiratory cycle into four phases, including end-inspiration, mid-expiration, mid-inspiration, and end-expiration.

After obtaining an absolute displacement of the heart in three dimensions (x-y-z) compared to a reference (for example, the first heartbeat), we then applied k-means clustering^39,40^ based on the three motion vectors to group all the heartbeats into multiple clusters, by assigning each data point to the cluster with the nearest mean and minimizing total intra-cluster variance. We used the default setting for initialization in the k-means clustering function, which was randomly choosing the initial seeds. We applied 10 times of the process to improve the convergence. Four clusters were chosen to span the respiratory motion from end-expiration to end-inspiration (and the two transition phases in between) to divide the data to four respiratory phases as described above. With the clustering, data at different heartbeats were assigned to the corresponding respiratory phases. Given the number of views per segment, the acquired readout lines were assigned to a series of cardiac phases based on the ECG triggers, for each of the respiratory phases.

### Data acquisition

The study was approved by the Institutional Review Board (IRB) at the University of California San Francisco (UCSF). Free-breathing 3D cardiac imaging was performed in 15 healthy volunteers (7 female, 8 males, age = 33.3 ± 7.6 years) in a short axis orientation on a 3.0 T MR scanner (GE Medical Systems, Milwaukee, WI) equipped with an 8-channel cardiac coil. Written, informed consent was obtained from all subjects prior to the MR scans. Relevant imaging parameters were: FOV = 34.0 × 25.5 cm^2^, slice thickness = 4.0–5.0 mm, image matrix = 256 × 144, number of slices = 28~32, TR/TE = 4.0–4.2/1.7 ms, flip angle = 60°, and readout bandwidth =  ± 125 kHz. CIRCUS (spiral pattern with parameter c = 1.5^25^) was applied with 75% partial Fourier along all three spatial dimensions (*k*_*x*_, *k*_*y*_ and *k*_*z*_). Averaged free-breathing 3D cine scan time was 2.5 ± 0.3 minutes. ECG leads and a respiratory bellow were placed to provide a record of cardiac and respiratory motion, respectively.

Multi-slice breath-hold 2D cardiac cine imaging was also acquired as the reference in 11 subjects using a balanced steady state free precession (bSSFP) sequence with the following imaging parameters: FOV = 34.0 × 25.5 cm2, slice thickness = 8 mm, image matrix = 224 × 144, number of slices = 12~15, TR/TE = 4.4/2.0 ms, flip angle = 60°, readout bandwidth = ±125 kHz, views per segment = 16, the number of reconstructed cardiac phases of 20, and imaging time per breath-hold/slice of ~11 seconds. Each slice was acquired during a single breath-hold (end-expiration) with prospective ECG gating. The total scan time for 2D imaging was 9.0 ± 2.3 minutes including resting between breath-holds (time for data acquisition itself was 2.6 ± 0.5 minutes).

### Image reconstruction

As described above, data were assigned to corresponding respiratory phases and cardiac phases to generate a 5D cardiac image-set. The number of views per segment for 3D data sets was chosen as 10 (corresponding to a temporal resolution of 10 × TR = 40–42 ms), and the number of reconstructed cardiac phases varied across different subjects with different heart rates. Cardiorespiratory-resolved 5D images were reconstructed using k-t SPARSE-SENSE that exploits joint multicoil sparsity along the cardiac dimension using finite differences to minimize total variation^16,41,42^. Image reconstruction was performed in MATLAB (The MathWorks, Natick, MA) and was implemented on a high-performance server with Four 2.5 GHz AMD Opteron 6380 CPUs and 256GB Memory. In this study, the image reconstruction took about 40 minutes reconstruction time per respiratory phase.

### Data analysis

Data clustering process and 5D image reconstruction have also been applied based on the previously developed 1D motion tracking^16^. Comparisons between 3D and 1D motion tracking were performed on the 15 human subjects, including evaluating the correlation between 3D and 1D motion curves, the dice coefficient of the clustering results, and the cardiac functional measurements.

The LV and RV volume-based parameters, including ESV, EDV, SV, and EF defined as (EDV-ESV)/EDV × 100, were measured in all datasets at both the end-expiratory and end-inspiratory phases. A customized software built in MATLAB was used to segment the LV and RV chambers^43^. This software enables automated segmentation in the 4D (3D + t) cardiac images by detecting the circular structures using the Hough transform and segmenting the LV using the circular structure in the proposed elliptically refined level set^43–46^. The Hough transform was used to automatically detect an initial circular-shape LV contour, followed by subsequent steps using an elliptically refined level set method to automatically segment the LV. During this process, the initial circular contour deforms to gradually fit the actual LV contour which is not required to be strictly circular. For the RV that has a more irregular shape, in this study we first generated an initial circular contour manually (centering and covering the whole RV), and then applied the same elliptically refined level set method to automatically detect the target RV contour. Regarding the segmentation at the base of the heart, we first manually decided which slices around the base to be included for ventricular volume calculation, and the above segmentation process was applied.

The functional measurements between end-expiration and end-inspiration for different genders, as well as those between 3D free-breathing and 2D breath-hold methods, were compared using a two-tailed paired-sample t-test, in which a *p*-value < 0.05 was considered statistical significance. The correlation coefficients between cardiac and respiratory related parameters were calculated, with a *p*-value < 0.05 indicating statistical significance. The Mann-Whitney test was applied to evaluate the difference in the measurements between female and male subjects.

Along with the cardiac measurements, we also measured respiration-induced parameters, including the respiratory rate, the three dimensional displacement of the heart between end-expiration to end-inspiration (corresponding to the respiratory depth, defined as Δd = [(c_1x_ − c_2x_)^2^ + (c_1y_ − c_2y_)^2^ + (c_1z_ − c_2z_)2]^1/2^, where [c_1×_, c_1y_, c_1z_] and [c_2×_, c_2y_, c_2z_] are the axis locations of the centroids of the two clusters), the percentage of the data from the entire respiratory cycle that occurs in end-expiration or end-inspiration phases, as well as the ratio between the end-expiration and end-inspiration percentages. We evaluated the correlations between the respiratory motion parameters and the cardiac function measurements.

## Supplementary information

Supplementary File

## Data Availability

The datasets generated during and/or analyzed during the current study are available from the corresponding author upon request. The source code for image reconstruction can be downloaded from http://cai2r.net/resources/software.
