# Supplementary material for: Extending Cardiac Functional Assessment with Respiratory-Resolved 3D Cine MRI
Source: Sci Rep. 2019 Aug 9;9:11563. doi: 10.1038/s41598-019-47869-z (PMC6689015; doi:10.1038/s41598-019-47869-z)
Supplement: Supplementary file 1 — Supplementary File [file 41598_2019_47869_MOESM1_ESM.zip › Supplements.docx]

**Extending Cardiac Functional Assessment with Respiratory-Resolved 3D Cine MRI**

Jing Liu^1^, Yan Wang^1^, Zhaoying Wen^1,2^, Li Feng^3^, Ana Paula Santos Lima^1^, Vaikom Mahadevan^4^, Ann Bolger^4^, David Saloner^1, 5^, Karen Ordovas^1^

**Affiliations**

^1^ Department of Radiology and Biomedical Imaging, University of California San Francisco, San Francisco, California, United States

^2^ Department of Radiology, Anzhen Hospital, Capital Medical University, Beijing, China

^3^ Department of Medical Physics, Memorial Sloan Kettering Cancer Center, New York, NY, United States

^4^ Department of Cardiology, University of California San Francisco, San Francisco, California, United States

^5^ Radiology Service, VA Medical Center, San Francisco, California, United States

**Address correspondence to:**

Jing Liu and Zhaoying Wen

185 Berry St, Suite 350

Radiology and Biomedical Imaging, University of California San Francisco

San Francisco, CA 94107

Tel: 415-514-8268 Fax: 415-353-9421

Email: [jing.liu@ucsf.edu](mailto:jing.liu@ucsf.edu), zhaoying.wen@ucsf.edu

**Supplement A 3D Motion Tracking versus 1D Motion Tracking**

To demonstrate the benefit of using 3D motion tracking versus 1D motion tracking, we have simulated the two motions involved in respiration: diaphragm and thoracic motion.

Figure SA1a shows the schematic diagram of the two orthogonal motion, diaphragm and thoracic motion, involved during the respiration. Figure SA1b demonstrates the thoracoabdominal asynchrony when there is phase angle between the two motion signals.

Figure SA2 shows the 2D display of the simulated diaphragm and thoracic motion with a series of phase angles in between them. Four assigned respiratory phases are highlighted in different colors, representing end-expiration, end-inspiration and the transition phases in between. Figure SA3a&b plots the data clustering for respiratory phases with 1D and 3D motion tracking respectively, for the series of phase angle between the diaphragm and thoracic motion. Note that to simplify the simulation, motion along the third dimension (z) was set to zero. Figure SA4 plots the accuracies of data clustering for the respiratory phases, by comparing the data clustering with 1D and 3D motion tracking (Figure SA3) and the reference (Figure SA2) respectively.

Our simulation results have demonstrated that 3D motion tracking provided more accurate data clustering of respiratory phases compared to the 1D motion tracking especially under thoracoabdominal asynchrony.


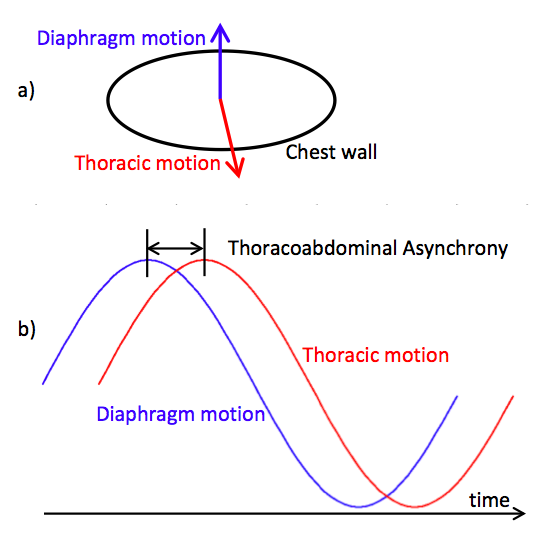


**Figure SA1**. Diaphragm and thoracic motion during respiration.


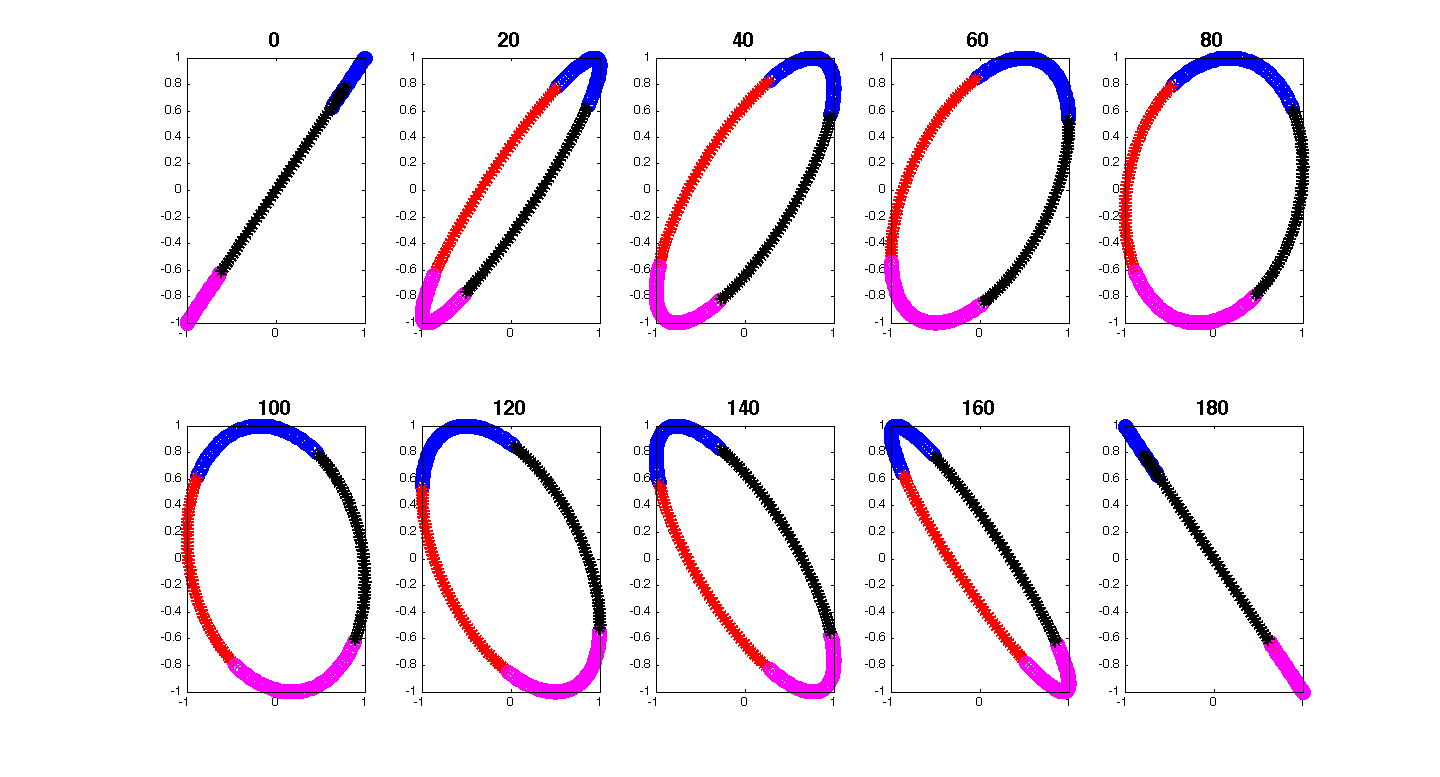


**Figure SA2**. Diaphragm and thoracic motion with different phase angles (from 0 to 180 degrees with an increment of 20 degrees).

**a)**
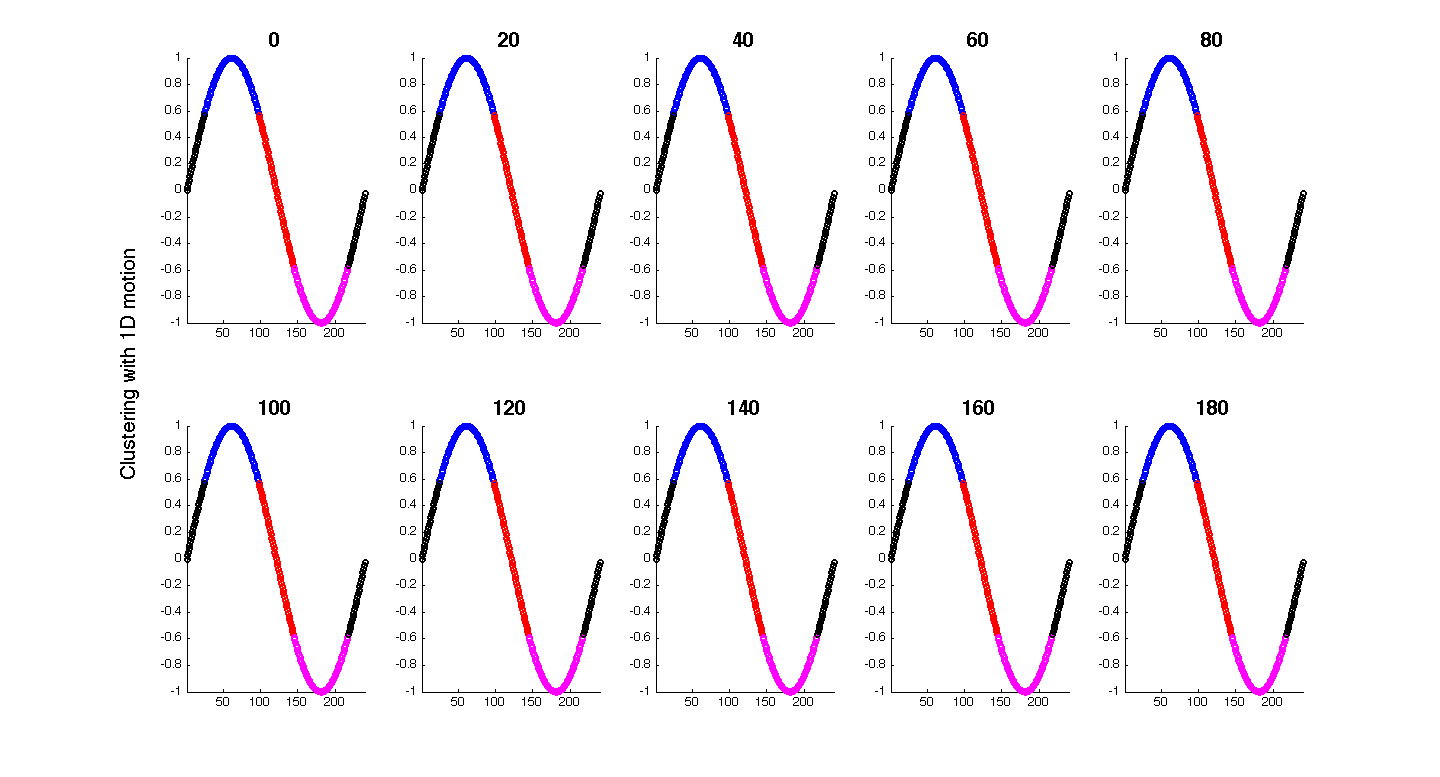


**b)**
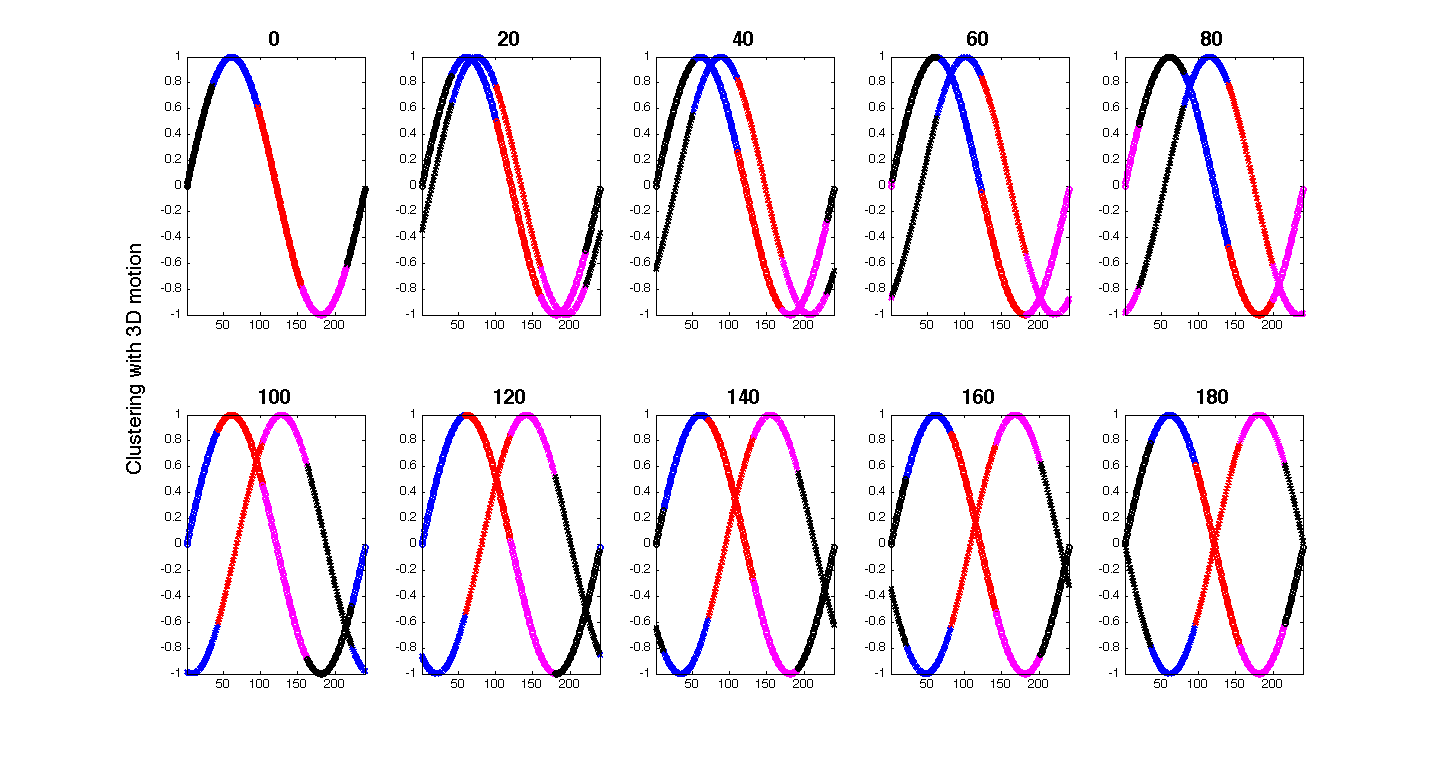


**Figure SA3**. Data clustering for respiratory phases with a) 1D motion tracking (diaphragm motion only) and b) 3D motion tracking (diaphragm and thoracic motion).


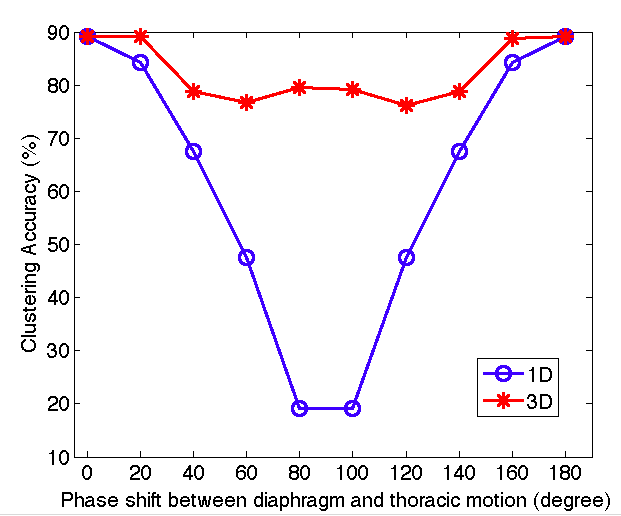


**Figure SA4**. Accuracies of the data clustering with 1D and 3D motion tracking.

**Supplement B Motion Tracking - Effect of Spatial Resolution**

We tested the effect of the spatial resolution on the motion tracking, based on simulated phantom data with a moving subject (mimicking the heart inside the chest).

Images with a moving subject were generated based on the simulated motion curves in two orthogonal directions (as shown in Figure SB1). Figure SB2 shows the simulated phantom with a subject moving anti-diagonally (motion curves from Figure SB1), where images at the two time points (#9 and #12 time points) are shown in a&b and the moving subject overlapped from the two image demonstrates the displacement between them. A movie of the simulated phantom is shown in d). Image resolution (1x1 mm^2^) and motion scale (-8~8 mm) were selected to be similar to those in the reality. Figure SB3 shows the simulated images of 1x1 mm^2^ resolution and those with lower resolutions, which were used for motion tracking respectively. Figure SB4 shows the estimated motion curves based on different image resolutions. Figure SB5 plots the mean errors of the estimated motion compared to the ground truth (reference). It demonstrated that motion tracking based on 2x2 or 4x4 mm^2^ image resolution provided reasonable accuracy. Figures SB6&7 shows results of the motion tracking with a smaller motion scale (-2~2 mm). It demonstrated that motion tracking based on image resolutions higher than 4x4 mm^2^ provided good accuracy.

The simulations demonstrated that the spatial resolution of 4x4 mm (used in our study) could provide reasonable motion tracking for images with resolution of 1x1 mm^2^, for either relatively large (-8~8 mm) or small (-2~2 mm) motion scales.


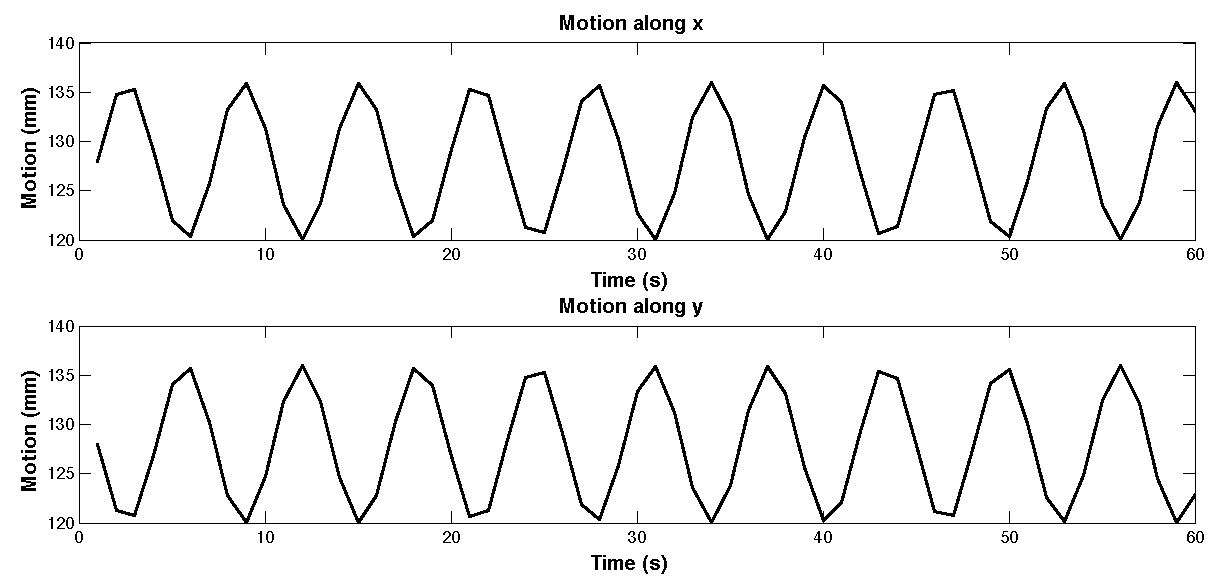


**Figure SB1**. Simulated motion curves along x and y directions, for motion range of -8~8 mm.


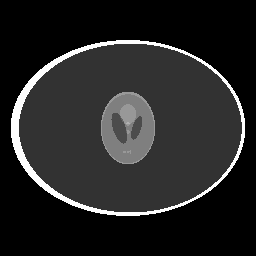


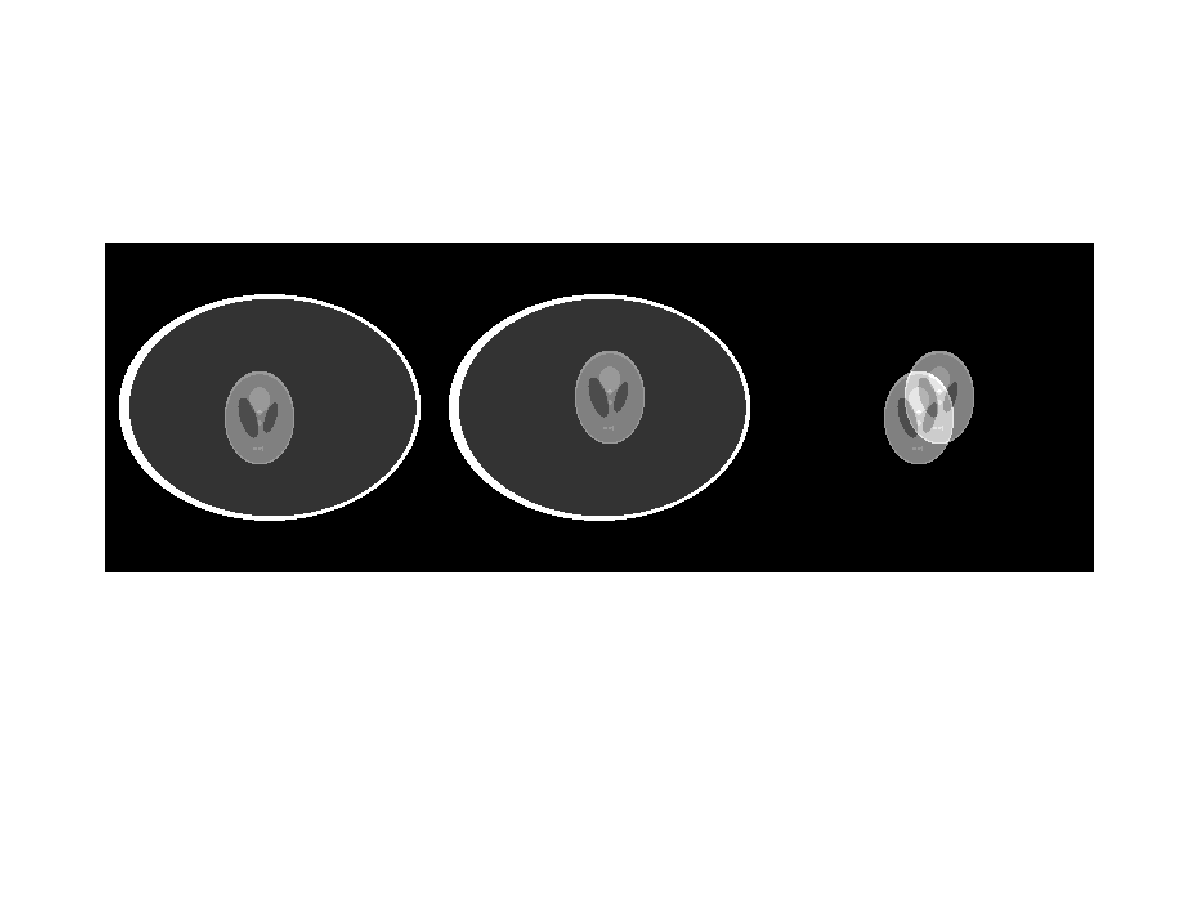


**a) b) c) d)**

**Figure SB2**. Simulated phantom with a moving subject. a&b): images at two representative time points; c): overlapped moving subject from a&b); d): movie of the simulated phantom with a moving subject. Motion is in the range of -8~8 mm.


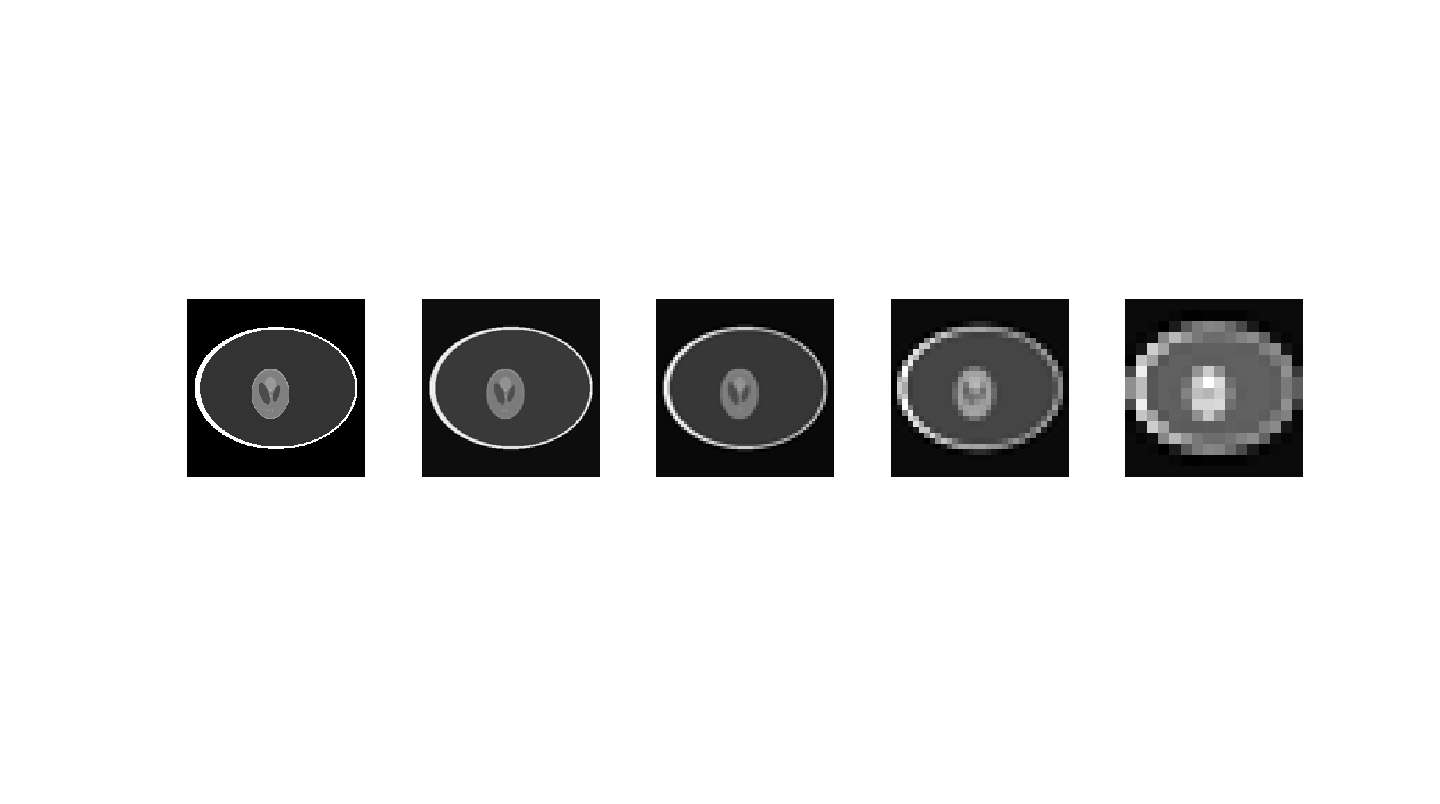


**Figure SB3**. Images with different spatial resolutions (from left to right: 1x1, 2x2, 4x4, 8x8, 16x16 mm^2^).


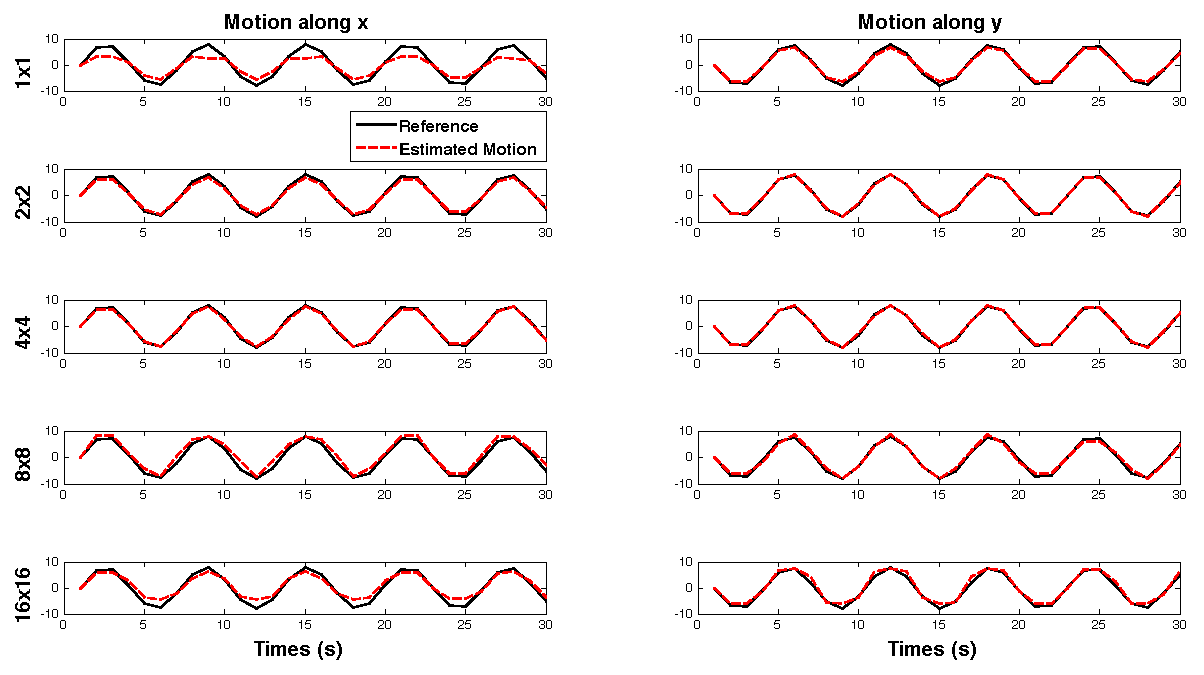

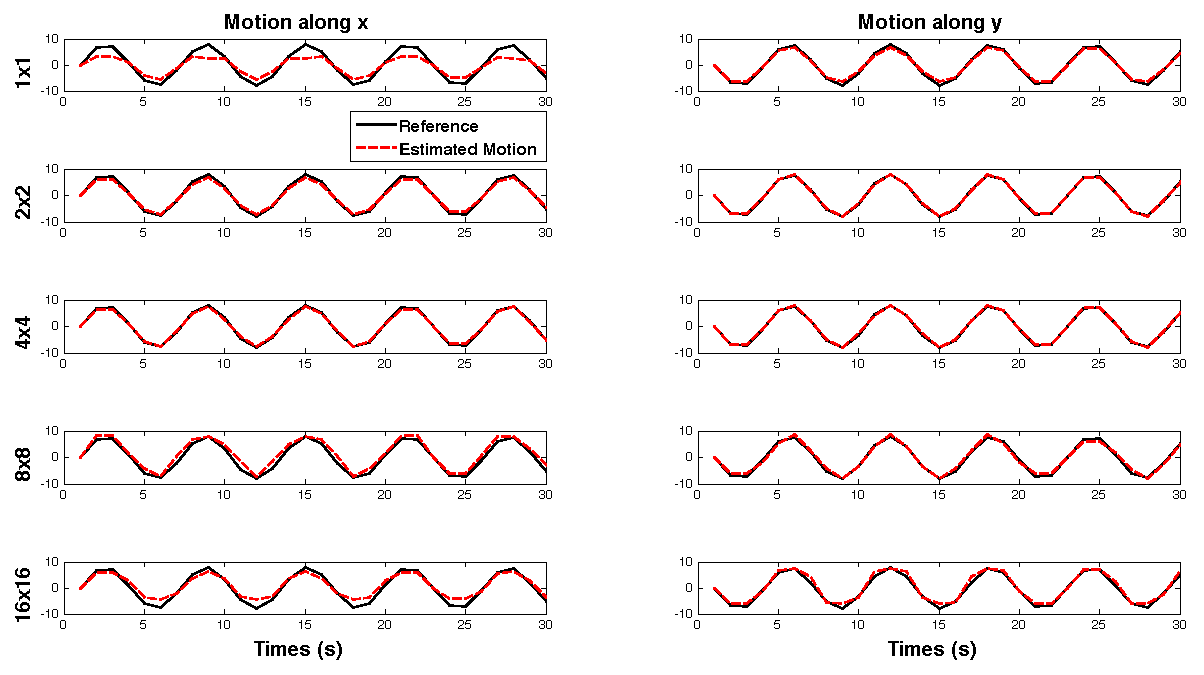


**Figure SB4**. Estimated motion curves based on different spatial resolutions, for motion range of -8~8 mm.


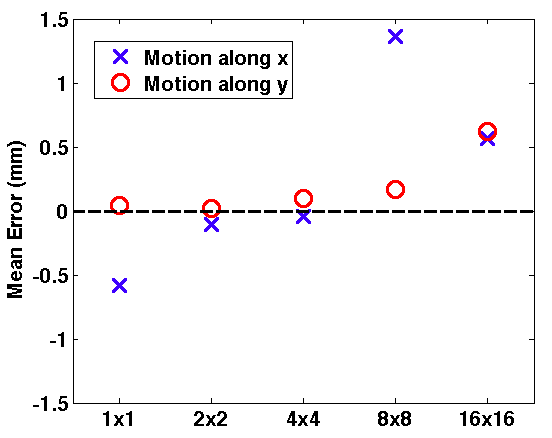


**Figure SB5**. Accuracies of the estimated motion curves based on different image spatial resolutions, for motion range of -8~8 mm.


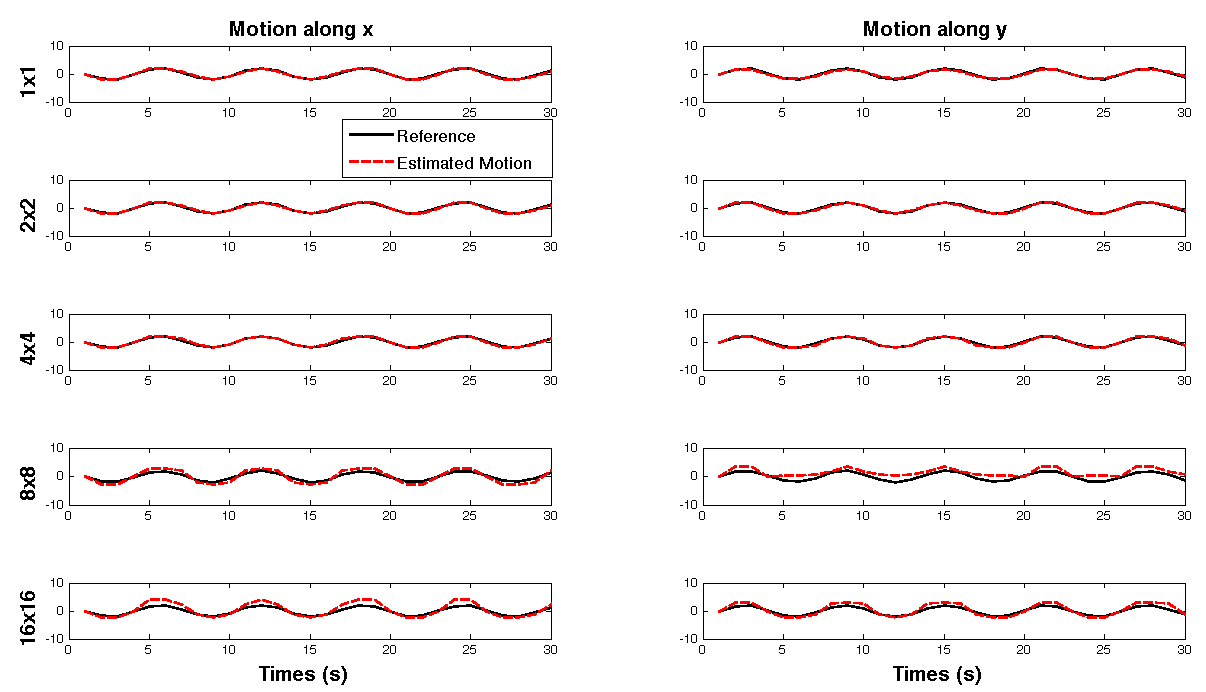

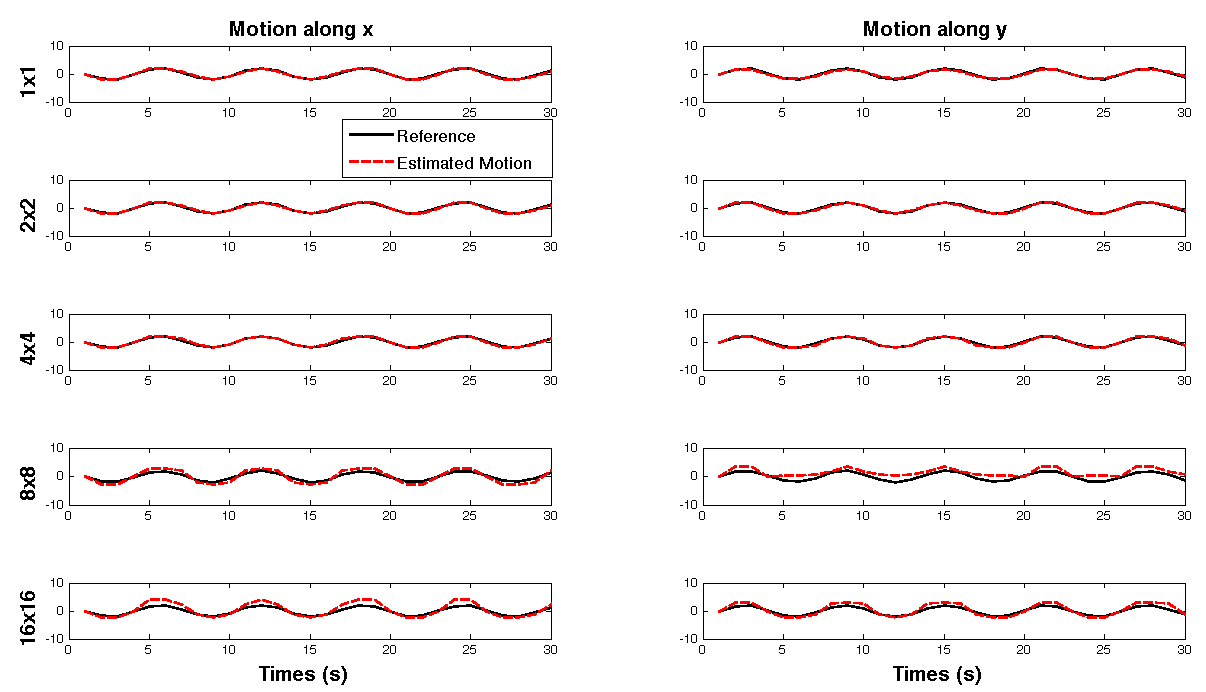


**Figure SB6**. Estimated motion curves based on different spatial resolutions, for motion range of -2~2 mm.


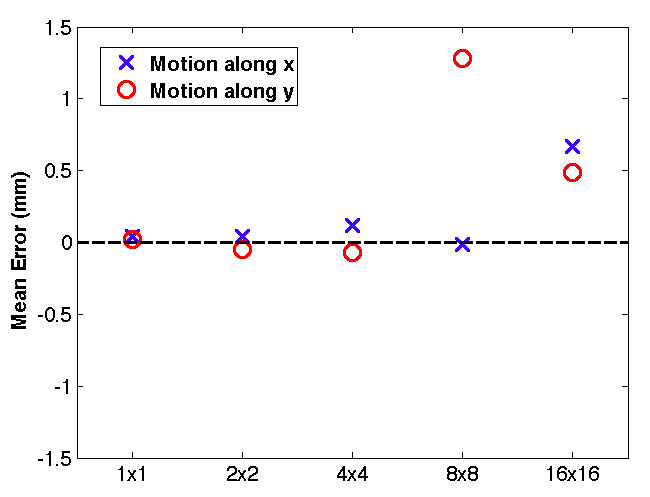


**Figure SB7**. Accuracies of the estimated motion curves based on different image spatial resolutions, for motion range of -2~2 mm.

**Supplement C Preliminary Patient Data**

We have applied the proposed 5D MRI in two patients with cardiac diseases (Figure SC1), including one (age 55 years, female) with atrial fibrillation and the other one (age 85 years, male) with aortic stenosis and pulmonary hypertension due to pulmonary fibrosis. The preliminary results from the two cardiac patients showed much larger changes in those cardiac functional measurements between end-expiration and end-inspiration. The changes were greatest in the patient with aortic stenosis and pulmonary hypertension: the LVESV, EDV, SV, and EF changed from end-expiration to end-inspiration by 15.7%, -9.1%, -22.7%, and -14.9%, respectively. This patient also had a larger respiratory depth (Δd=8.9 mm vs 5.3±2.3 mm in volunteers), as well as larger average motion within the data during end-expiration (4.8 mm vs 1.9±1.3 mm in volunteers) and end-inspiration (3.8 mm vs 2.8±2.5 mm in volunteers). This suggests that tracking the respirophasic effects on the heart with 5D imaging may be important in accurately and reproducibly assessing cardiac dysfunction, and CMR restricted to a single respiratory phase may not accurately reflect the actual range of cardiac performance over the respiratory cycle.


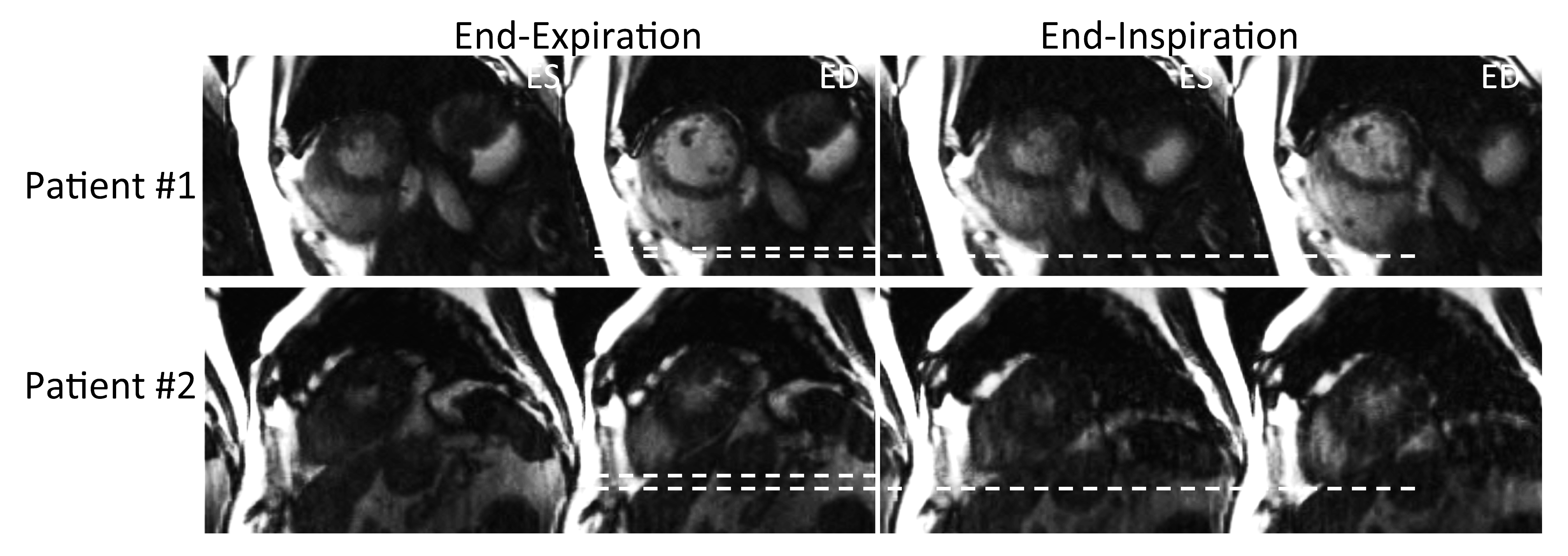


**Figure SC1**. Images from patients with arrhythmia (top row) and aortic stenosis and pulmonary hypertension (bottom row) respectively. The dotted lines highlight the respiratory depths along readout direction.
